# From challenges to solutions: Strengthening mental health support for university students and young researchers in China

**DOI:** 10.1002/gps3.70021

**Published:** 2026-05-06

**Authors:** Yuqian Chen, Shaohua Hu

**Affiliations:** ^1^ Department of Psychiatry the First Affiliated Hospital Zhejiang University School of Medicine Hangzhou Zhejiang China; ^2^ Nanhu Brain‐Computer Interface Institute Hangzhou Zhejiang China; ^3^ Zhejiang Key Laboratory of Precision Psychiatry Hangzhou Zhejiang China; ^4^ MOE Frontier Science Center for Brain Science and Brain‐Machine Integration Zhejiang University School of Medicine Hangzhou Zhejiang China; ^5^ Brain Research Institute of Zhejiang University Hangzhou Zhejiang China; ^6^ The State Key Laboratory of Brain‐Machine Intelligence Zhejiang University Hangzhou Zhejiang China; ^7^ Zhejiang Engineering Center for Mathematical Mental Health Hangzhou Zhejiang China

In the past two decades, China has gained global recognition for rapid development in higher education and scientific research. Meanwhile, these achievements come with intense—and often less visible—psychological pressures for university students and young researchers. Throughout this correspondence, ‘university students’ refers to undergraduates and students pursuing advanced degrees, whereas ‘young researchers’ refers to research‐track master's and doctoral students, research assistants and postdoctoral researchers, typically in their early 20s to mid‐30s, across fields such as medicine, engineering, life sciences and social sciences. This is not a marginal group, given that the number of graduate students in China increased from 1.85 million in 2014 to 4.09 million in 2024, and the number of students recruited for higher education has continued to rise in recent years.^1^ Though the authorities and administration have gradually recognised the importance of mental health for these populations, the current status and strategies remain understudied.

The Blue Book of Mental Health: Report on National Mental Health Development in China (2023–2024) indicates that depressive symptoms peak in the 18–24 age group, and anxiety levels are higher in urban populations. These findings position university‐age youth as a high‐risk population for mental ill‐health. Such evidence shows that poorer mental health aligns with lower academic resilience, underscoring the need for early intervention in educational settings.^2^

University students face multiple stressors, including academic workload, interpersonal challenges and uncertainty in a competitive labour market. University students must navigate uncertain employment prospects and the immense pressure to assemble a portfolio of grades, awards, experiences and internships.^3^ Stigma and low mental health literacy may delay help‐seeking. At the same time, logistical barriers such as wait times, out‐of‐pocket costs or limited campus capacity can decrease enrolment in care and increase dropout once care has begun.^4^

Research‐track students encounter additional profession‐specific stressors, including long working hours, publication pressure and insecure career trajectories. Career uncertainty is a major part of this burden because the rapid expansion of research training has not been matched by a similar growth in stable academic positions. Left unaddressed, these stressors may lead to burnout and mental disorders, undermining both well‐being and scientific innovation.^5^ Evidence further shows that burnout among Chinese medical students is associated with compromised empathy, professional identity and untoward mental health.^6^

A multi‐faceted strategy is therefore needed. Firstly, at the individual level, mental health should be conceptualised as a long‐term responsibility similar to the management of chronic conditions. Young adults should be encouraged to proactively monitor their well‐being, adopt protective lifestyle practices and seek help spontaneously. Such routine self‐care aligns with the *Healthy China 2030 Planning Outline*, which emphasises cultivating autonomous health behaviours.^7^ Normalising self‐monitoring and timely support will reduce the lag between the onset of first symptoms and seeking care. However, obstacles remain for many young adults in recognising symptoms, overcoming stigma and accessing support. Therefore, clear, straightforward information is needed to help young people name their experiences, decide when to seek care and understand which support resources are available. Such psychoeducation should be sensitive and supportive, emphasising that mental health problems are common, treatable and worthy of attention, thereby helping youth to feel more protected from stigma or privacy invasion while seeking help.

Secondly, at the technological level, digital and intelligent tools can provide new opportunities. Some recent work emphasises the growing role of digital mental health interventions in addressing service gaps and expanding support within China's mental health system.^8^ As a clinical example, an AI‐assisted diagnostic model named *Emoface* has been built to analyse dynamic facial signals to differentiate bipolar disorder from major depressive disorder, reaching high diagnostic accuracy in clinical testing.^9^ *Emoface*'s case reflects the potential of AI‐assisted diagnostic approaches to reduce barriers to appropriate care, particularly when integrated into structured clinical pathways. Meanwhile, other wearable devices—such as brain–computer‐interface products designed to monitor neural activity—may help young adults obtain timely feedback and prompt low‐threshold self‐care. Several local governments have been supporting the development and implementation of such technologies. Users' major concerns are potential data insecurity and a lack of privacy protection; therefore, safeguards for privacy, equity and ethics are fundamental and indispensable. At the same time, digital tools should complement, rather than replace, professional clinical assessment. Their value may lie in selected settings, such as psychoeducation, freshman orientation and targeted support for higher‐risk students, where they can assist early identification and referral. Over‐reliance on digital tools alone may delay treatment in severe cases.

Thirdly, at the institutional level, mental health cannot be the sole responsibility of hospitals or psychiatric professionals. For example, universities could develop a stepped system. Initially, basic mental health education could be integrated into student orientation and courses; then, targeted support could be offered for higher‐risk groups such as first‐years and research trainees in high‐demand labs; next, mentors and teachers could be trained to spot early warning signs among their students and access referral pathways; finally, a sustained connection should be maintained between the institution and the counselling and psychiatry departments of local hospitals. A recent scoping review underscored the importance of comprehensive systems that integrate psychoeducation, screening, referral and combined interventions.^10^ Additionally, universities and research institutes could establish formal training programmes for research‐track trainees, including topics such as burnout prevention, boundary setting and management of publication pressure and potential academic rejection, such as unsuccessful manuscript submissions and programme applications. Barriers to help‐seeking, such as uneven institutional capacity, confidentiality concerns and fear of academic repercussions, can be mitigated only if protections are not merely stated but actively enforced. This requires protected gatekeeper time, an explicit privacy policy with the strict separation of health and academic records, and an institutional policy that guarantees the absence of academic penalty for seeking mental health assistance.

Lastly, at the policy level, institutional and national frameworks could place greater emphasis on the well‐being of young researchers and students by reducing reliance on narrow performance indicators, such as publication counts, journal prestige and grant income. More secure career pathways could be supported through transparent promotion criteria, stable contract arrangements and clearer transition routes. Universities, funding agencies and government should all recognise that psychological well‐being is essential for sustainable academic productivity. For example, institutions could allocate dedicated funding for preventive well‐being programmes or include mental‐health support capacity in institutional accreditation standards, which could be used to evaluate whether universities provide adequate services. Supportive policies from the Ministry of Education, the National Health Commission and funding agencies could provide the structural foundation necessary for real change. For young researchers specifically, actions such as expanding funding schemes or providing alternative academic and nonacademic career pathways are likely to reduce chronic psychological strain. Progress may be slowed by the entrenched evaluation culture that persists in China, limited budgets and the fragmented oversight responsibilities for mental health that rest with educational and health care providers. Even so, national attention to mental health is rising, creating a favourable climate for coordinated action and turning local pilots into standard practice.

China's young generation embodies both the promise and the pressure of the nation's rapid development. Although university students and young researchers share the same higher‐education environment, the latter face an additional layer of research‐specific pressure that deserves separate attention. Protecting their mental health is therefore not only an ethical imperative but also a strategic investment for the country's future. This correspondence highlights the mental health needs of university students and young researchers as a shared yet heterogeneous population and proposes the need to integrate individual, technological, institutional and policy‐level responses, as illustrated in figure 1. Such coordinated, system‐level action is urgently needed.

**FIGURE 1:**
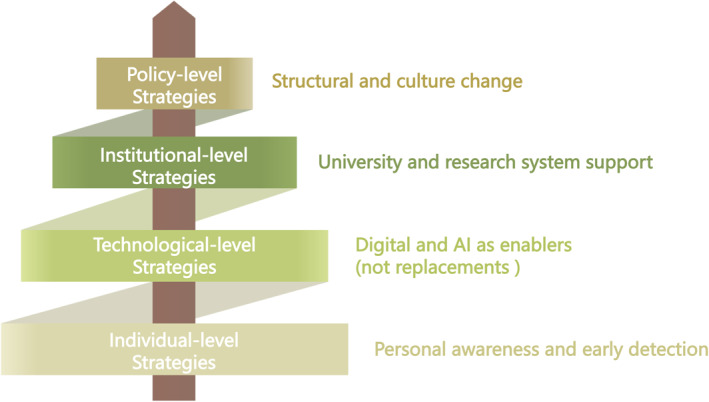
Mental health support for Chinese university students and young researchers requires a coordinated, multi‐level system: empowering individuals, strengthening institutions, reforming policies and leveraging technology as an enabling tool.

## AUTHOR CONTRIBUTIONS

**Yuqian Chen**: Conceptualization; literature review; writing. **Shaohua Hu**: Conceptualization; supervision; editing.

## FUNDING

This work received no specific funding.

## CONFLICT OF INTEREST STATEMENT

The authors declare no conflicts of interest.

## ETHICS STATEMENT

This correspondence does not report original human or animal research and therefore does not require ethical approval.

## Data Availability

No new data were generated or analysed in this study. Data sharing is not applicable.
